# The Relationship Between School-Based Research and Preschool Teachers’ Teaching Ability: The Mediating Role of Constructivist Beliefs in Teaching

**DOI:** 10.3389/fpsyg.2022.814521

**Published:** 2022-03-15

**Authors:** Zhonglian Yan, Shoumei Zhao

**Affiliations:** Faculty of Education, Northeast Normal University, Changchun, China

**Keywords:** school-based research, teaching ability, teaching belief, the mediating role, preschool teacher professional development

## Abstract

To understand the relationship between kindergarten school-based research (SBR) and preschool teachers’ teaching ability and identify the mechanism by which SBR affects teachers’ teaching ability, a survey of randomly sampled preschool teachers in Sichuan Province (*N* = 625, M_age_ = 26.61) was conducted online using three scales assessing community learning, teaching ability and teaching philosophy. The survey results showed that the level of SBR reached the upper-middle level. The level of SBR positively predicted the teaching beliefs and teaching ability of preschool teachers. SBR influenced teaching ability through direct and indirect effects, with indirect effects accounting for 23.5% of the total effect; however, only constructivist teaching beliefs played a mediating role in the relationship between SBR and teaching ability *via* an indirect effect. SBR focused on improvement in teaching ability should not only examine the connection between SBR and practical problems to promote improvement in teachers’ teaching ability but also should examine the role of teachers’ beliefs and influence teachers’ teaching abilities by changing teachers’ teaching beliefs.

## Introduction

High-quality education is the pursuit of contemporary education (Early et al., 2007; Tikly and Barrett, 2011). Teacher competence is considered a key factor affecting the quality of education (Fauth et al., 2019; Manning et al., 2019). The professional development of teachers has been regarded as a key way to ensure the quality of education (Tatto, 2015). According to *professional learning community* theory, teachers’ professional development should be closely related to the classroom teaching situation, and it should implemented through teachers’ individual reflection and peer coaching in professional learning community (Imants et al., 2001; Shulman and Sherin, 2004; Singer and Moscovic, 2008). SBR directly affects the improvement of teachers’ teaching ability due to the emphasis on practical problems (Gao and Wang, 2014), and it is regarded by researchers as an effective approach to teachers’ professional development (van Velzen and Volman, 2009; Fang, 2017). In addition, SBR changes teachers’ teaching beliefs by continuously promoting teachers’ reflection (de Vries et al., 2013; Chaaban, 2017) and, in turn, leads to improvement in teachers’ teaching abilities. There are two ways to influence teachers’ teaching abilities: direct influence and indirect influence. *Via* indirect effects, teachers’ teaching beliefs play a mediating role. Therefore, studying the relationships among SBR, teaching beliefs and teaching abilities is conducive to further confirming the value of SBR.

### School-Based Research as a Learning Community

School-based research in kindergarten in China is also related to the conduct of school-based curricula. The wave of school-based curriculum reform in China in 2001 promoted the exploration of theory and practice of the curriculum for children in kindergarten (Chen and Yan, 2001; Li, 2006). Because of limitations of preschool teachers’ quality, curriculum development cannot be carried out in many kindergartens, and SBR in kindergarten has been proposed by some Chinese researchers on ECE (Zhang and Zhu, 2005; Zhu and Wang, 2005).

School-based research in kindergarten involves preschool teachers, teaching and research managers, teaching and research experts and other relevant parties in the teaching and research community. Actively paying attention to the kindergarten care and teaching process and solving the actual problems of early childhood education (ECE) and care can improve the quality of care and ECE and promote the professional development of teachers. Research and practice are parallel activities for the ultimate goal of child development (Huang, 2019). It has been proposed that the nature of SBR is action research, which originated from the “Teacher Action Research Movement” in the 1950s. Commonality is reflected in multi-agent participation, that is, the use of science by teachers, administrators and other relevant personnel. Some methods of research aim to improve practice (Martyn, 1993). Since SBR in kindergarten is often focused on a certain problem (such as the curriculum), SBR usually involves workshops. Therefore, SBR is essentially a “learning community” organized for kindergartens (Huang, 2017).

School-based research is regarded as a professional learning community (PLC) (Shulman and Sherin, 2004; Huang, 2017). A PLC is a school organization structure with an intellectually directed culture typified by “reflection, dialog, sharing, and practice.” According to their common interests or goals, the members share, inspire, explore and apply to achieve common vision and development goals through collective learning (Louis and Marks, 1998; Molla and Nolan, 2019).

### School-Based Research Is Conducive to Improving Teachers’ Teaching Ability

Due to the situational nature of teaching activities, although teachers already possess relevant theoretical knowledge of education and teaching, the application of this knowledge in practice must be combined with specific scenarios, and SBR is the most suitable way to encourage teachers to improve their teaching ability (Imants et al., 2001; Chen and Zhang, 2014).

However, other studies suggest that the relationship between SBR (i.e., teacher professional development workshops) and changes in teachers’ teaching behavior ability is still uncertain (Schachter, 2015). Because SBR in kindergarten focuses on practical problems in kindergartens and pays attention to the educational situation of children and kindergartens (Li, 2011), and SBR in kindergarten should promote improvement in preschool teachers’ teaching ability. Therefore, this research proposes hypothesis H1 as follows: SBR can positively predict preschool teachers’ teaching ability.

### School-Based Research Is Conducive to Changing Teachers’ Teaching Beliefs

Teaching ability is a professional ability that promotes the achievement of teaching goals based on professional and pedagogical knowledge in a specific educational and teaching situation. It represents the teaching and organizational ability of a teacher in teaching and education activities (Dios et al., 2018). The potential for teaching to be improved by SBR is mainly reflected in the fact that teachers can acquire practical knowledge, promote the contextualization of existing knowledge, and acquire specific behaviors and ways of doing things (Chen and Zhang, 2014).

Teachers’ beliefs involve their views concerning the curriculum and the relationship between teachers and students (Peterson et al., 1989). Teachers’ teaching beliefs include two aspects, namely, traditional teaching beliefs and constructivist teaching beliefs (CTBs). Traditional teaching beliefs refer to teacher-centered beliefs; teachers with such beliefs often pay more attention to the results of learning rather than the process. In contrast, CTBs are reflected in student-centered beliefs and are focused on cultivating students’ critical thinking, reflective ability, and cooperative ability (Teo and Zhou, 2017; Papadakis and Kalogiannakis, 2020; Papadakis, 2021).

Chan and Elliott (2004) compiled the Teaching Belief Scale (TLCQ), which is divided into two dimensions, namely, traditional teaching beliefs and CTBs. The scale has 30 items in total. A simplified version of the Conceptions of Teaching and Learning (COTL) scale was developed by Teo and Zhou (2017). The scale also includes two dimensions: traditional teaching concepts (TTCs) and constructivist teaching concepts (CTCs). For this research, the simplified Teachers’ Teaching Beliefs Scale compiled by Teo and Zhou was adopted.

Changing teachers’ beliefs requires the presentation of the problem situation and the teacher’s own reflection (Yang, 2010) and SBR, which emphasizes practical problems and essentially presents the real problem situation; thus, the reflective environment of the teacher community naturally drives teachers’ individual reflection. SBR can therefore change the beliefs of teachers.

Generally, participating in teaching and research activities (especially centralized education and training) can help teachers adopt advanced educational concepts (Pang and Ye, 2000). The cultivation of teacher education concepts needs to be based on the teacher’s experience and the school education situation (Liang and Xin, 2015), which are characteristics of SBR. The research of de Vries et al. (2013) showed that teachers’ participation in professional development activities (participation in seminars, training and other activities to update knowledge and skills) has an important impact on their teaching beliefs. For teaching activities among teachers with the same background, reflection and criticism among teachers is also conducive to changes in teachers’ educational concepts. Chaaban (2017) confirmed through research that SBR can change teachers’ educational concepts. This view is similar to related peer learning (Cui and Wang, 2016) and apprenticeship training (Rinke et al., 2014; Billett, 2016).

Some researchers have also found that professional learning communities are conducive to the development of preschool teachers’ teaching beliefs (Keung et al., 2020); that is, school-based training or SBR that values practical experience is conducive to improving teachers’ beliefs (Hursen and Islek, 2017). However, some scholars argue that the relationship between the two is not clear (Lander et al., 2017). Therefore, this research attempts to verify the relationship between SBR and teachers’ educational beliefs and proposes Hypothesis H2 as follows: SBR has a positive predictive effect on teachers’ teaching beliefs.

### The Relationship Between Teachers’ Teaching Beliefs and Teaching Ability

Teachers’ teaching behavior is influenced by their beliefs (Yu, 2007; Mertala, 2019; Papadakis and Kalogiannakis, 2020); teachers’ teaching beliefs are the internal foundation of all teachers’ thoughts, words and deeds in the classroom, including the presentation of the subject content, the presentation method, and students’ difficulty in mastering the learning content.

Xin et al. (1997) studied the relationship between the educational beliefs of primary school teachers and their teaching monitoring ability and found a significant correlation between them; that is, teachers’ educational beliefs directly affected their teaching monitoring ability. Once a teacher’s teaching monitoring ability was improved, the teacher consciously change inappropriate teaching behaviors.

Preschool teachers’ beliefs have been found to have a greater impact on their ability (Mohamed and Al-Qaryouti, 2016; Kim, 2017). Teachers with stronger beliefs show more positive practical intentions (Yin et al., 2021). Since educational beliefs and educational abilities may have a consistent theory and foundation, only combining the two in practice can truly promote improvement in teachers’ quality (Pang and Ye, 2000).

Huang and Zhang (2019) found a weak correlation between preschool teachers’ beliefs in preschool mathematics education and their teaching behaviors through a survey of 120 preschool teachers. However, a survey of 274 preschool teachers by Sim and Miai (2021) showed a significant positive correlation between the teaching ability and teaching beliefs of preschool teachers. Different numbers of survey respondents usually produce different conclusions. This study attempts to expand the sample size and further examine the relationship between preschool teachers’ teaching beliefs and teaching abilities. In particular, it emphasizes the predictive effect of teaching beliefs on teaching ability. Therefore, hypothesis H3 is proposed as follows: the teaching beliefs of preschool teachers have a positive predictive effect on their teaching abilities.

If the previous three hypotheses are correct, preschool teachers’ teaching beliefs may play an intermediary role between SBR and preschool teachers’ teaching ability. Therefore, hypothesis H4 is proposed: preschool teachers’ teaching beliefs play an intermediary role between SBR and teaching ability.

### Value of the Research

In the high school stage, teachers’ participation in SBR activities is conducive to improving teachers’ teaching ability (Wei et al., 2021). Does the same relationship exist the ECE stage? Some researchers believe that participating in SBR can change teachers’ teaching beliefs and promote teachers’ teaching ability. In other words, teachers’ educational beliefs have an intermediary effect between SBR and teachers’ educational ability. Currently, there is no relevant quantitative research to support this statement. The existing research has been from the perspective of teacher participation (Ke et al., 2019), but quantitative research on teacher participation in SBR is relatively lacking.

The importance of the diversity of kindergarten classes for the implementation of the ECE curriculum has been emphasized. However, in China, there are not only age differences in kindergarten students but also differences between kindergartens (Chen and Yan, 2001; Li, 2006). Therefore, the curriculum should be developed based on the diversity and specialty of each kindergarten. In the opinion of researchers, SBR can improve teachers’ teaching ability (Chen and Zhang, 2014; Dios et al., 2018), but there is a lack of quantitative research on this issue. Thus, the study attempts to use quantitative research to illustrate the relationship among SBR, teaching beliefs, and teaching ability.

In addition, since SBR is essentially a type of cultural learning, studying the relationship between SBR and teachers’ teaching concepts and teaching ability is conducive to further confirming the value of SBR based on quantitative research and is beneficial for further exploring the mechanism by which SBR affects teaching ability.

## Materials and Methods

### Participants

The participants were *N* = 625 preschool teachers (female, M_age_ = 26.61) recruited online in Sichuan, People’s Republic of China, using Questionnaire Star. A total of 653 questionnaires were collected, and 625 valid questionnaires were obtained, with an effective rate of 95.7%.

A total of 2.2% of preschool teachers had an advanced teacher job title, 8% had a first-level teacher job title, 14.7% had a secondary-level teacher job title, 5.8% had a third-level teacher job title, and 69.3% had an unrated job title. A total of 1.8% of preschool teachers had earned a postgraduate degree, 93.6% had earned a bachelor’s degree or had completed junior college, and 4.6% had completed high school.

### Procedure

The survey procedures in the study were reviewed and approved by the Ethics Review Board of Northeast Normal University. The survey was completed online by 653 preschool teachers in Sichuan Province in China who voluntarily participated in the questionnaire. The questionnaire survey was divided into two processes: a pilot survey and a formal survey. There were 100 preschool teachers participating in the pilot survey. The data from the pilot survey showed that the reliability and validity of the questionnaire were good and that a formal questionnaire survey could be conducted.

### Instruments

#### School-Based Research Level

The Dimensions of the Learning Organization Scale (DLOQ Scale) (Watkins and Marsick, 2003) was accepted in the PLC (Louis and Marks, 1998; Dou and Zhang, 2006). This study adopted the PLC scale compiled by Louis and Marks (1998) and revised by Yin et al. (2019). As such, preschool teachers completed the *sense of purpose* (SP) subscale, which comprises 3 items (e.g., “Most of my colleagues agree with me on what the developmental goals of kindergarten should be,” α = 0.89); the *collective focus on student learning* (CFSL) subscale, which comprises 4 items (e.g., “My colleagues and I will provide children with a variety of suitable activities,” α = 0.96); the *reflective dialog* (RD) subscale, which comprises 4 items (e.g., “During brainstorming sessions, we discuss the methods and strategies for assessing young children’s behavior,” α = 0.97); the *deprivatized practice* (DPR) subscale, which comprises 3 items (e.g., “My colleagues often observe the performance of the children in our class,” α = 0.93); and the *cooperative activity* (CA) subscale, which comprises 5 items (e.g., “I often conduct teaching and research activities with my colleagues,” α = 0.94) rated on a 5-point scale (from 1 = “strongly disagree” to 5 = “strongly agree”). The PLC has been shown to be reliable and valid in Chinese teachers (Yin et al., 2019).

#### Preschool Teachers’ Teaching Belief

This study adopted the Conceptions Of Teaching and Learning (COTL) scale developed by Teo and Zhou (2017). As such, preschool teachers completed the *traditional teaching beliefs* subscale, which comprises 5 items (e.g., “The effect of teacher’s direct teaching is better than that of children’s self-exploration,” α = 0.97), and the *constructivist teaching beliefs* subscale, which comprises 5 items (e.g., “Young children should be given many opportunities to express their ideas,” α = 0.91) rated on a 7-point scale (from 0 = “strongly disagree” to 6 “strongly agree”). The COTL has been shown to be reliable and valid (Teo and Zhou, 2017).

#### Preschool Teachers’ Teaching Ability

In this study, we used *the Scale of Organizational and Didactic Competencies for Educators* (ESCOD) compiled by Dios et al. (2018). As such, preschool teachers completed the *teaching* subscale, which comprises 14 items (e.g., “Adjust the activity plan according to the education and teaching situation,” α = 0.93), and the *organization* subscale, which comprises 9 items (e.g., “Collaborate with kindergarten colleagues and leaders,” α = 0.89) rated on a 7-point scale (from 0 = “no development” to 6 “very well developed”). The ESCOD has been shown to be reliable and valid (Dios et al., 2018).

### Analytical Strategy

In this study, SPSS 25.0 was used to conduct *t test*s, correlation analyses and regression analyses of the three variables. Then, we used AMOS 23.0 to analyze the fit indices of the scales and the mediating role of preschool teachers’ teaching beliefs in the relationship between SBR and teachers’ teaching ability. The fit indices of the scales (X^2^/df, IFI, CFI, NFI, RFI, TLI, RMSEA, Table 4) in the study were within the acceptable range (Hu and Bentler, 1999), indicating that the model had good fit and good structural validity.

**TABLE 1:** The difference test of SBR and teaching ability.

	Variable		*N*	Mean	SD	F/*t* value	Comparison afterward
TA	K-R	D- kindergarten	155	5.80	1.15	3.25*	4 < 1*; 5 < 1*, 3*
		F- kindergarten	78	5.68	1.27		
		S- kindergarten	157	5.68	0.98		
		T-kindergarten	27	5.27	1.21		
		U- kindergarten	208	5.42	1.20		
TA	E-B	Master’s degree	11	4.80	1.53	2.16	
		University	585	5.62	1.20		
		High school	29	5.74	1.10		
TA	J-T	A- teacher	14	6.03	0.84	3.91**	3 < 2*; 5 < 2*
		F-teacher	50	6.15	1.03		
		S- teacher	92	5.57	0.96		
		T- teacher	36	5.73	1.17		
		U- teachers	433	5.53	1.19		
TB	K-R	D- kindergarten	155	4.70	1.34	1.61	
		F- kindergarten	78	4.47	1.38		
		S- kindergarten	157	4.52	1.31		
		T-kindergarten	27	4.71	0.99		
		U- kindergarten	208	4.37	1.23		
TB	E-B	Master’s degree	11	3.82	1.25	2.19	
		University	585	4.54	1.29		
		High school	29	4.28	1.35		
TB	J-T	A-teacher	14	4.60	1.05	0.31	
		F- teacher	50	4.67	1.24		
		S- teacher	92	4.57	1.19		
		T- teacher	36	4.47	1.54		
		U- teachers	433	4.49	1.31		
SBR	K-R	D- kindergarten	155	4.14	0.83	4.02**	5 < 1*, 2*, 3*
		F- kindergarten	78	4.13	0.85		
		S- kindergarten	157	4.11	0.74		
		T- kindergarten	27	3.84	0.69		
		U- kindergarten	208	3.86	0.81		
SBR	E-B	Master’s degree	11	3.47	0.97		
		University	585	4.04	0.79	2.81	
		High school	29	3.95	1.06		
SBR	JT	A- teacher	14	4.38	0.63	2.48*	3 < 2* 5 < 2*
		F- teacher	50	4.28	0.66		
		S- teacher	92	3.98	0.74		
		T- teacher	36	4.13	0.85		
		U- teachers	433	3.98	0.83		

*p < 0.05; **p < 0.01, same following.

*K-R, kindergarten rank; E-B, educational background; J-T, job title; TA, teaching ability; TB, teaching belief; D, demonstration; A, advanced level; F, first-level; S, secondary-level; T, third-level; U, unrated; High school (including vocational and technical secondary school); University (including junior college and undergraduate); Master’s degree, Master’s degree and above.*

**TABLE 2:** The correlation matrix of SBR, teaching beliefs, and teaching abilities.

variable	M	SD	J-T	Age	1	2	3	4	5	6	7	8	9	10	11
J-T	–	–													
Age	26.61	–	–	–											
1	4.02	0.81	−0.10*	0.09*	–										
2		0.89	−0.12**	0.12**	0.86**	–									
3		0.91	−0.05	0.05	0.93**	0.79**	–								
4		0.88	−0.13**	0.11**	0.91**	0.74**	0.79**	–							
5		0.88	−0.06	0.05	0.88**	0.67**	0.79**	0.74**	–						
6		0.90	−0.08*	0.08	0.92**	0.71**	0.80**	0.81**	0.82**	–					
7	4.51	1.29	−0.04	−0.03	0.39**	0.35**	0.35**	0.33**	0.42**	0.35**	–				
8	3.38	2.04	0.01	−0.09*	0.14**	0.13**	0.14**	0.07	0.22**	0.11**	0.90**	–			
9	5.93	1.26	−0.10*	0.12**	0.62**	0.54**	0.52**	0.62**	0.52**	0.59**	0.49**	0.06	–		
10	5.61	1.15	−0.13*	0.17**	0.64**	0.53**	0.55**	0.60**	0.57**	0.62**	0.33**	0.10*	0.57**	–	
11	5.49	1.16	−0.12**	0.17**	0.62**	0.52**	0.53**	0.57**	0.56**	0.60**	0.34**	0.12**	0.54**	0.98**	–
12	5.74	1.20	−0.13**	0.16**	0.63**	0.52**	0.54**	0.60**	0.56**	0.61**	030**	0.06	0.56**	0.97**	0.91**

*p < 0.05; **p < 0.01, 1 = SBR (total); 2 = SP; 3 = CA; 4 = CFSL; 5 = DPR; 6 = RD; 7 = TB (total); 8 = TTC; 9 = CTC; 10 = TA (total); 11 = T; 12 = O.

**TABLE 3:** Regression analysis results of SBR level, teaching ability and teaching beliefs (*N* = 625).

Dependent variable	Independent variable	B (Unstandardized regression coefficient)	SE (Standard error)	Adjusted R^2^	ΔR^2^	β (Standardized regression coefficient)	*t*
TAs	SBR	0.91	0.04	0.41	0.41	0.64	20.65***
TB	SBR	0.63	0.06	0.15	0.16	0.39	10.71***
TAs	TB	0.29	0.03	0.11	0.11	0.33	8.65***
	TTB	0.03	0.02	0.32	0.32	0.06	1.82
	CTB	0.52	0.03			0.56	16.99***

***p < 0.001.

**TABLE 4:** The fit index.

Index	X^2^/df	RMSEA	CFI	NFI	RFI	TLI	IFI
Good	≤3	≤0.08	≥0.9	≥0.9	≥0.9	≥0.9	≥0.9
Acceptable	≤5	≤0.10	≥0.8	≥0.8	≥0.8	≥0.8	≥0.8
SBR	2.70	0.09	0.96	0.93	0.92	0.95	0.97
Teaching ability	2.48	0.09	0.97	0.95	0.95	0.97	0.97
Teaching belief	2.80	0.09	0.97	0.96	0.95	0.97	0.97
Mediating model	4.9	0.8	0.99	0.98	0.97	0.98	0.97

## Results

### Preliminary Analyses

Descriptive statistics and correlations for all study variables are displayed in Tables 1, 2. The results from F/*t* tests indicate no differences in teaching beliefs by job title (*M*_advanced–level_ = 4.60, *SD* = 1.05; *M*_first–level_ = 4.67, *SD* = 1.24; *M*_secondary–level_ = 4.57, *SD* = 1.19; *M*_third_ _–level_ = 4.47, *SD* = 1.54; *M*_unrated_ = 4.49, *SD* = 1.31; *F* = 0.31, *p* > 0.05). There is a significant difference in SBR by teachers’ job titles (*M*_advanced–level_ = 4.38, *SD* = 0.63; *M*_first–level_ = 4.28, *SD* = 0.66; *M*_secondary–level_ = 3.98, *SD* = 0.74; *M*_third_ _–level_ = 4.13, *SD* = 0.85; *M*_unrated_ = 3.98, *SD* = 0.83, *F* = 2.48, *p* < 0.05) and kindergarten rank (*M*_Demonstration–k_ = 4.14, *SD* = 0.83; *M*_first–level–k_ = 4.13, *SD* = 0.85; *M*_secondary–level–k_ = 4.11, *SD* = 0.74; *M*_third_ _–level–k_ = 3.84, *SD* = 0.69; *M*_unrated_ = 3.86, *SD* = 0.81, *p* = 0.31, *F* = 4.02, *p* < 0.01), and a difference in teaching ability between by teachers’ job titles (*M*_advanced–level_ = 6.03, *SD* = 0.84; *M*_first–level_ = 6.15, *SD* = 1.03; *M*_secondary–level_ = 5.57, *SD* = 0.96; *M*_third_ _–level_ = 5.73, *SD* = 1.17; *M*_unrated_ = 5.53, *SD* = 1.19, *F* = 3.91, *p* < 0.01).

Furthermore, there is a significant difference in teaching ability by kindergarten rank (*p* < 0.05) and no differences in teaching ability by teachers’ educational backgrounds (*p* > 0.05) (Table 1).

Table 2 shows that the overall level of SBR in the kindergartens where the survey respondents were located is at the upper-middle level (*M*_SBR_ = 4.02 > 3, *SD* = 0.81), and the overall level of preschool teachers’ teaching ability is at the upper-middle level (*M*_TA_ = 5.61 > 3, *SD* = 1.15).

### Correlation Analyses

Based on the linear correlation analysis of SBR in kindergarten and preschool teachers’ teaching ability (Table 2), the overall level of SBR and its dimensions are significantly positively correlated with preschool teachers’ teaching ability and its dimensions (*p* < 0.01). The overall level of SBR has a moderately positive correlation with preschool teachers’ teaching ability and its various dimensions. The correlation coefficient with total teaching ability is 0.64.

The linear correlation analysis between the level of SBR and teaching beliefs (Table 2) shows that SBR and all its dimensions have a significant positive correlation with preschool teachers’ teaching beliefs and its dimensions (*p* < 0.01). The correlation coefficient between the overall level of SBR and teaching beliefs is 0.39.

The linear correlation analysis of teaching beliefs and teaching ability (Table 2) shows no correlation between traditional teaching beliefs and teachers’ organizational ability and a significant positive correlation between other factors (*p* < 0.01). The correlation coefficient between preschool teachers’ teaching beliefs and teaching ability is 0.33.

### Regression Analysis

Under the premise that SBR is significantly positively correlated with preschool teachers’ teaching ability, we further test the interpretation and prediction of the effect of the level of SBR on preschool teachers’ teaching ability. In this study, the SBR level and its various dimensions (i.e., sharing goals, cooperative activities, focus on children’s learning and development, sharing practices, and reflective dialog) were used as independent variables, preschool teachers’ teaching ability was used as the dependent variable, and the forced input method was used to perform linear regression analysis.

Table 3 shows that in the regression model of SBR level and teaching ability, the adjusted R^2^ value is 0.41, indicating that the predictive power of SBR level on preschool teachers’ teaching ability reaches 41%. At the same time, the value of the standardized coefficient β is 0.64 (a positive value), and the value of t is 20.65 (*p* < 0.001), indicating that the level of SBR has an extremely significant positive predictive effect on preschool teachers’ teaching ability. For every additional unit level of SBR, preschool teachers’ teaching ability increases by 0.638 units. Therefore, H1 is verified.

Table 3 shows that in the regression model of the SBR level and teaching beliefs, the adjusted R^2^ value is 0.15, indicating that the predictive power of the SBR level on preschool teachers’ teaching beliefs is 15%. At the same time, the value of the standardized coefficient β is 0.39 (a positive value), and the value of t is 10.71 (*p* < 0.001), indicating that the level of SBR has an extremely significant positive predictive effect on preschool teachers’ teaching beliefs, and for every additional unit of SBR, preschool teachers’ teaching beliefs increase by 0.39 units. Therefore, H2 is verified.

Table 3 shows that in the regression model of teaching beliefs and teaching ability, the adjusted R^2^ value is 0.11, indicating that the predictive power of preschool teachers’ teaching beliefs on preschool teachers’ teaching ability is 11%. Furthermore, the value of the standardized coefficient β is 0.33 (the value is a positive number), and the value of t is 8.65 (*p* < 0.001), indicating that preschool teachers’ teaching beliefs have an extremely significant positive predictive effect on their teaching ability. For every additional unit of teaching beliefs, teaching ability increases by 0.33 units. Therefore, H3 is verified.

In addition, in the regression model of each dimension of teaching beliefs and teaching ability, the adjusted R^2^ value is 0.32, indicating that the predictive power of preschool teachers’ teaching beliefs on their teaching ability reaches 32%. The value of the standardized coefficient β of CTBs is 0.56 (a positive value), and the t value is 16.99 (*p* < 0.001), indicating that CTBs can significantly predict preschool teachers’ teaching ability, while the predictive effect of traditional teaching beliefs on preschool teachers’ teaching ability does not reach a significant level. Specifically, for every additional unit of CTBs, preschool teachers’ teaching ability increases by 0.56 units.

### Test of the Mediating Effect

From the above correlation analysis and regression analysis, it can be seen that the level of SBR, preschool teachers’ teaching beliefs and their teaching ability have significant effects (hypotheses H1, H2, and H3 in this research are supported). Furthermore, the SBR level and teaching beliefs play a significant positive role in predicting preschool teachers’ teaching ability. In view of this, we explore whether the level of SBR in kindergarten directly affects preschool teachers’ teaching ability through preschool teachers’ teaching beliefs, which indirectly affect their teaching ability, to further understand the process by which the level of SBR affects preschool teachers’ teaching ability. Based on hypothesis H4, this research tests the mediating effect of preschool teachers’ teaching beliefs on the relationship between the SBR level and preschool teachers’ teaching ability.

Because traditional teaching beliefs and CTBs among preschool teachers are variables with opposite directions, the two dimensions of preschool teachers’ teaching beliefs are established to test the mediation effect. The results show that (1) the mediating effect of traditional teaching beliefs on the SBR level and preschool teachers’ teaching ability is not significant (the confidence interval includes zero) and (2) the mediating effect of CTBs on the SBR level and preschool teachers’ teaching ability is significant (the confidence interval does not include zero). The mediating effect of CTBs is as follows.

**FIGURE 1:**
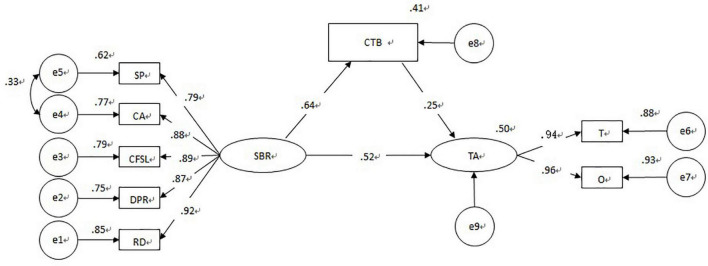
A model diagram of the mediating effect of CTBs.

To test the mediating effect of CTBs in preschool teachers’ teaching beliefs on the SBR level and preschool teachers’ teaching ability, this study uses Amos 23.0 to take the SBR level and its various dimensions as independent variables. Taking preschool teachers’ teaching ability and its various dimensions as dependent variables and taking the constructivist dimension of preschool teachers’ teaching beliefs as mediating variables, we construct a structural equation model (as shown in Figure 1) to test the mediating effect. According to the specific operating procedures, the sample size is set to 5,000, the confidence interval is set to 95%, and the calculation is performed. The analysis of the CTB mediation model shows that it has a good fit (Table 4).

First, with regard to the SBR level in kindergarten, preschool teachers’ teaching beliefs and teaching ability models (Table 5), the total effect between the SBR level and preschool teachers’ teaching ability is 0.68, and the Z value is 16.54, which is significantly greater than 1.96. The bias-corrected 95% confidence interval (CI) is (0.60, 0.75), and the percentile 95% CI is (0.60, 0.75). Neither contains 0, indicating that the total effect of the SBR level on kindergarten and preschool teachers’ teaching ability is significant.

**TABLE 5:** The mediating role of the CTBs bootstrap test (SBR—TAs).

	Point estimate	Product of coefficient		Bias-corrected 95% CI		Percentile 95% CI	
Effect		SE	Z	Lower	Upper	Lower	Upper
Total effect	0.68	0.04	16.54	0.60	0.75	0.60	0.75
Indirect effect	0.16	0.04	3.88	0.09	0.25	0.09	0.25
Direct effect	0.52	0.05	9.61	0.41	0.62	0.41	0.62

***p < 0.001.

Second, the indirect effect of preschool teachers’ CTBs between the SBR level and preschool teachers’ teaching ability is 0.16; the Z value is 3.88, which is significantly greater than 1.96; and the bias-corrected 95% CI is (0.09, 0.25). The percentile 95% CI is (0.09, 0.25), and neither contains 0, indicating that preschool teachers’ CTBs have a significant indirect effect between the SBR level in kindergarten and preschool teachers’ teaching ability.

Third, after controlling for the influence of teaching beliefs in the intermediate variables, the direct effect of the SBR level and preschool teacher’s teaching ability is 0.52, and the Z value is 9.61, which is significantly greater than 1.96. The bias-corrected 95% CI confidence interval is (0.41, 0.62), and the 95% CI is (0.41, 0.62), neither of which contains 0, indicating that the direct relationship between the level of SBR and preschool teachers’ teaching ability is significant.

Finally, preschool teachers’ CTBs have a mediating effect between the SBR level and preschool teachers’ teaching ability (hypothesis H4 in this study is partially established), and the indirect effect accounts for 23.5% of the total effect. That is, the level of SBR directly affects teaching ability and indirectly affects teaching ability by affecting preschool teachers’ teaching beliefs.

In summary, of the four research hypotheses in this study, H1, H2, and H3 are verified, and H4 is partially verified (Figure 1); that is, the SBR level, teaching beliefs and teaching ability are all significantly correlated, and preschool teachers’ CTBs played an intermediary role between SBR and preschool teachers’ teaching ability. Therefore, the level of SBR not only directly affects preschool teachers’ teaching ability but also indirectly affects teaching ability by influencing teachers’ CTBs.

## Discussion and Analysis

### The School-Based Research Level Has Been Greatly Improved

In this study, the level of kindergarten-based teaching and research reached the upper-middle level (*M* = 4.02), which is different from the “low efficiency and formality of kindergarten-based teaching and research” and “poor level of kindergarten-based teaching and research” mentioned by some researchers (Li, 2006; Liu, 2010; Lu, 2011). In approximately 2005, due to the low quality of kindergarten teachers and the overall low level of kindergarten management, the level of kindergarten-based teaching and research was low. However, after 15 years of development and the standardization of the kindergarten management system, the quality of kindergarten-based teaching and research have been ensured. In particular, the quality of kindergarten teachers has greatly improved, and kindergartens themselves have been equipped with high-level teaching and research staff. This teaching and research involves a higher level of learning community.

First, SBR is based on the existence of a certain community (Zhang and Zhu, 2005; Huang, 2017). Currently, SBR is generally equipped with a higher-quality learning community. Researchers suggest that many preschool teachers in China have not previously received professional training (Pang, 2019). For example, among preschool teachers in Beijing in 2010–2015, nearly 40% of young teachers had a technical secondary school education or below (Feng et al., 2017). In 2013, the preschool teachers in Sichuan Province were similar to this level. In Sichuan Province, the percentage of full-time preschool teachers with a high school education and below is 32.33%, and 67.67% of preschool teachers have a university degree or above, which means that more than 30% of preschool teachers do not meet the educational standard.

With the attention of the Chinese government, the number of preschool teachers in China has steadily increased, and the professional level has improved (Yu and Zhang, 2018), which is mainly reflected in the great improvement of preschool teachers’ academic qualifications and profession (Gao and Wang, 2014).

In 2020, there were 2,913,400 full-time preschool teachers in China, an increase of 150,300 over the previous year (5.44% increase) (Ministry of Education in China, 2020). The ratio of teachers with a high school degree and below was 14.25%, which is less than 20%, and 80% of teachers had a college degree or above.

In 2020, the number of full-time preschool teachers in Sichuan Province with a high school degree or below dropped to 9.39% (13876/147849). That is, more than 90% of preschool teachers had a college degree or above (90.61%). In this study, the proportion of preschool teachers with a college degree or above accounted for more than 90% (Table 1). Among all the teachers, preschool teachers majoring in preschool education accounted for 88.3%. The improvement in the quantity and quality of preschool teachers provides a good foundation for SBR.

Second, the standardized management of kindergartens provides a guarantee for the quality of SBRs. Since 2010, China has issued many policies and documents related to ECE to standardize and guide the development of kindergartens and to ensure the quality of ECE, such as “Qualification Standards for Principals,” “Professional Standards for Preschool Teachers,” and “Guidelines for the Development of Children from 3 to 6 Years Old.” In the “Kindergarten Work Regulations,” various safety systems for kindergarten operations are clearly proposed. Local governments have also equipped professional management personnel, such as county-level preschool education teachers and researchers, to ensure business exchanges in county kindergartens. The “Criteria for the Appointment of the Principal” strengthens admission standards to ensure the normal operation of kindergartens. The “Kindergarten On-the-Job Training Curriculum Standards” and “Several Opinions of the State on the Reform of Early Childhood Education” clearly propose requirements for teacher on-the-job training and SBR. The forthcoming “Preschool Education Legislation Draft” also proposes standards for SBR. In approximately 2005, researchers felt that SBR in kindergarten was inefficient and formalized (Li, 2006; Liu, 2010; Lu, 2011). After years of hard work, with the standardization of the kindergarten management system, the quality of SBR in kindergarten has been ensured.

School-based research is more standardized in the selection and training of principals than teachers. The “Qualification Standards for Principals” have been issued in China to strengthen the barriers to entry for principals. At the same time, these standards attach importance to the training of principals and key teachers and the quality of training courses. As the government attaches emphasizes the training of principals and expert teachers, the problem awareness and research capabilities of principals and key teachers have been significantly improved (Jiao, 2020). Although there is still theoretical content in the training, the training also includes analysis of the problems of the kindergarten and the kindergarten’s future development plan. The principals and expert teachers who receive the training pay more attention to the problems of the kindergarten and the practice of theories in ECE. During the training, visits to different types of kindergartens and reflection and dialog with different principals promote principals’ reflective thinking and open consciousness. In this context, the guidance of county-level teachers and researchers promotes exchanges between different kindergartens in the county and provides a basis for demonstration and research for the in-depth study of problems in kindergartens.

Third, changes in the form and content of SBR have improved the effectiveness of SBR. In the process of China’s educational development, teachers’ continuing education has always been valued. Especially since 2010, a diverse preschool teacher training system with rich content has been formed in China, including workshops, SBR, and a coaching system. Training attaches great importance to the participation of preschool teachers (Huang, 2017). This series of changes highlights the central position of teachers in training (Zhu and Chang, 2018). In particular, SBR focusing on practical problems in kindergartens has transformed preschool teachers from passive learners in traditional training to researchers, reflectors, and learning leaders. The role of preschool teachers has undergone a fundamental change (Chen, 2017), and the dominant position of kindergarten teachers in SBR has been highlighted.

Guidance from theoretical knowledge is critical to training for preschool teachers, but more importantly, such training pays attention to the connection between theory and practice and the solution of practical problems. In particular, researchers reflect on the content of SBR and the effects of training to promote training at all levels and to promote SBR to emphasize practical knowledge and practical problems in kindergartens (Li, 2011). Training for kindergartens clearly shows two trends: the first involves increasing the connection with practice in kindergartens and promoting the connection between theory and practice in kindergartens, while the second involves increasing reflection and improvement.

Under the guidance of local preschool researchers and driven by expert teachers and model kindergartens, exchanges among county-based preschool teachers have formed a regular system, and expert preschool teachers have the courage to show others their practice and exploration in ECE. This provides a good research atmosphere for SBR. The promotion of the famous teacher studio system has deepened the connection between ECE theory and kindergarten practice and promoted an atmosphere of reflection and discussion in all kindergartens. After 2015, the expert teacher studio system was launched in various places. On the one hand, it has strengthened the connection among kindergartens and promoted the construction of the kindergarten learning and practice community. On the other hand, the expert teacher studio system has focused on the practice of kindergartens and strengthened the relationship between theory and practice. Effective convergence, to a certain extent, also provides a model for SBR. The expert teacher studio system focuses on the practice of kindergartens and strengthens the effective connection between theory and practice while providing a model for SBR in each kindergarten.

In addition, in various workshops led by expert teachers, preschool teachers encounter problems in practice as the research content. Through the “community” in the workshops, preschool teachers and local kindergartens can think collaboratively. Opportunities for practical contact increase opportunities for joint reflection and improve the various abilities of participating teachers. Under the guidance of expert teachers, preschool teachers become good at speaking, reflecting, and innovating.

### The School-Based Research Level Can Positively Predict Teaching Beliefs and Teaching Abilities

According to *professional learning community* theory, SBR that closely reflects the actual kindergarten situation can change teachers’ beliefs (Imants et al., 2001; Shulman and Sherin, 2004). Since the real problems of kindergartens involve the content of their teaching and research activities to promote solutions to the actual problems of ECE (Wu, 2021), SBR draws upon teachers’ previous experiences, increases teachers’ recognition of research and training, promotes teachers’ reflection, and enhances teachers’ teaching beliefs. Furthermore, it promotes the improvement of preschool teachers’ teaching ability, which shows a positive correlation between SBR, teaching beliefs and teaching ability, supporting the views of some researchers (de Vries et al., 2013; Chen and Zhang, 2014; Chaaban, 2017; Hursen and Islek, 2017; Kim, 2017; Yin et al., 2019; Keung et al., 2020; Sim and Miai, 2021).

However, these findings are inconsistent with the views of some studies. Some researchers believe that the SBR level is relatively low and cannot promote a change in preschool teachers’ beliefs and an improvement in teaching behavior (Li, 2006; Liu, 2010; Lu, 2011), which should be related to the SBR level or working mechanism. The advantage of ideal SBR is the sharing of practical situations (Chen and Zhang, 2014; Liang and Xin, 2015). It is difficult to transform teaching concepts into teaching abilities, but when combined with school practice, teachers’ experience can be utilized, and teachers can transform their concepts into self-monitoring abilities (Xin et al., 1997). Therefore, the function of SBR should be established based on shared practice. Only by combining the actual shared practice of ECE in kindergarten can teaching beliefs be transformed into teaching behaviors.

### In the Context of Shared Goals and Cooperative Activities, Teaching Beliefs Can Positively Predict Teaching Ability

Since the reform of ECE in China is basically guided by constructivist theory, the focus on the central position of children and the emphasis on the value of play causes SBR to attach importance to the subjective status of children and the role of play to further deepen teachers’ CTBs (Louis and Marks, 1998). Additionally, because the content of SBR combines the practical problems of kindergartens, preschool teachers can organically combine teaching beliefs with teaching practice. Similarly, some researchers believe that teaching ability and teaching beliefs are significantly positively correlated (Mohamed and Al-Qaryouti, 2016; Kim, 2017; Sim and Miai, 2021).

This view is different from the weak correlation that some researchers have found (Schachter, 2015; Huang and Zhang, 2019). Huang and Zhang (2019) suggested that there is a weak correlation between teaching beliefs and teaching behaviors. The main reason for this is that teaching behavior and teaching ability are not the same concept, and their focuses are different. Teaching ability in this study mainly focuses on teaching and organization, while teaching behaviors involve emotional support, class management, and educational support (Huang and Zhang, 2019). Since the teaching behavior of preschool teachers is affected not only by preschool teachers’ own ability but also by the kindergarten environment and other factors, whether teaching ability is completely transformed into teaching behavior remains to be further studied.

### Constructivist Teaching Beliefs Play a Part in the Mediating Role Between SBR and Preschool Teachers’ Teaching Behaviors

Researchers believe that school-based training can effectively change teachers’ beliefs due to problematic situations and teachers’ reflections (Yang, 2010). In child-centered and play-centered curricula, teachers’ constructivist beliefs have a positive effect (Sim and Miai, 2021). This positive effect is mainly reflected in the impact on behavior (Mohamed and Al-Qaryouti, 2016; Kim, 2017). Therefore, SBR guided by constructivist theory can change the teaching beliefs of preschool teachers and generate changes in teaching behavior. In this study, the indirect effect of preschool teachers’ constructivist beliefs between SBR and teaching ability accounted for 23.5% of the total effect (Table 5), indicating that teachers’ teaching beliefs played a mediating role between SBR and teaching ability. The direct impact of SBR on the teaching behavior of preschool teachers was 76.5%. Traditional teaching beliefs have no mediating effect on the relationship between SBR and teachers’ teaching ability.

## Conclusion and Suggestions

### Conclusion

School-based research is a systematic project that focuses on the core of the quality of ECE, which emphasizes play as the main form of learning and the central position of children (Teo and Zhou, 2017; Papadakis and Kalogiannakis, 2020; Papadakis, 2021), develops a series of research forms and systems, and provides a good foundation for the development of SBR to improve the level of SBR.

(1) SBR in China has reached the upper-middle level. There is a diverse preschool teacher training system with rich content in China, including workshops, SBR, and a coaching system.

In such a teacher training system, due to the great importance attached to the guiding role of experts and local officials and the emphasis on kindergarten practice, SBR has played an important role in promoting the professional growth of teachers and the construction of the kindergarten curriculum, and it has shown a high level.

(2) The SBR level can positively predict preschool teachers’ teaching beliefs and teaching abilities. In China, SBR focusing on practical problems in kindergartens has transformed preschool teachers from passive learners in traditional training to researchers, reflectors, and learning leaders, enabling teachers to think about practice in ECE. Preschool teachers’ research ability and habits have improved. When teachers apply these abilities to practice, they continue to improve their teaching ability. These reflective habits and the democratic atmosphere formed in workshops are transferred to SBR, which drives more teachers to participate.
(3) The constructivist beliefs of preschool teachers play an intermediary role between SBR and teaching ability. The impact of SBR on teachers’ teaching ability occurs in two ways: indirect and direct impacts. In this study, the direct impact of SBR on the teaching behavior of preschool teachers accounted for 76.5% of the total effect, and the indirect effect accounted for 23.5%; preschool teachers’ constructivist beliefs played a mediating role in the relationship between SBR and teaching ability *via* an indirect effect.

### Suggestions

Since there are two mechanisms by which SBR affects preschool teachers’ teaching ability, there are two ways to promote teachers’ teaching abilities.

(1) SBR should be developed based on the practice of ECE in kindergartens to promote improvement in the teaching ability of kindergarten teachers.
(2) The role of preschool teachers’ beliefs should be considered; the purpose of influencing preschool teachers’ teaching abilities can be achieved by changing teachers’ teaching beliefs.

### Implications

There are still some deficiencies in this study, especially in terms of the methods. First, the subjects of the questionnaire were only from one province in China; thus, the study’s representativeness is not sufficient, and the sample size is relatively limited. Second, only questionnaires were used, and no interviews were conducted with relevant preschool teachers or principals. Third, in terms of the research design, this study was only a cross-sectional study and not a longitudinal controlled experiment, indicating that the research method was not perfect.

## Data Availability Statement

The data and material used during the current study are available from the corresponding author (ZY) on reasonable request.

## Author Contributions

ZY and SZ were responsible for the design of the study. ZY was responsible for writing and revision of the manuscript. SZ was responsible for the data analysis. Both authors contributed to the article and approved the submitted version.

## Conflict of Interest

The authors declare that the research was conducted in the absence of any commercial or financial relationships that could be construed as a potential conflict of interest.

## Publisher’s Note

All claims expressed in this article are solely those of the authors and do not necessarily represent those of their affiliated organizations, or those of the publisher, the editors and the reviewers. Any product that may be evaluated in this article, or claim that may be made by its manufacturer, is not guaranteed or endorsed by the publisher.
